# Severe bilateral isolated coronary ostial lesions as a rare manifestation of radiation-induced cardiac disease A case report

**DOI:** 10.1097/MD.0000000000009867

**Published:** 2018-03-30

**Authors:** Metesh Nalin Acharya, Mohammad El-Diasty, Bastian Schmack, Alexander Weymann, Ashham Mansur, Aron-Frederik Popov

**Affiliations:** a Department of Cardiothoracic Surgery, Harefield Hospital, Harefield, UK; b Department of Cardiac Surgery, University Hospital Oldenburg, Oldenburg; c Department of Anesthesiology, University Medical Centre, Georg August University, Goettingen; d Department of Cardiac Surgery, University Hospital Frankfurt, Frankfurt, Germany.

**Keywords:** cardiac surgery, coronary, irradiation, ostium

## Abstract

**Rationale:**

With advances in contemporary radiotherapy techniques, and as cancer survival improves, severe isolated coronary ostial disease may develop many years following mediastinal radiotherapy, even in the absence of classical cardiovascular risk factors.

**Patient concerns:**

We describe the case of a 73-year-old woman with previous chest radiotherapy for breast cancer who underwent coronary artery bypass graft surgery for severe bilateral coronary ostial lesions.

**Diagnoses:**

Coronary angiography demonstrated severe, isolated bilateral coronary ostial lesions.

**Interventions:**

The patient underwent urgent coronary artery bypass graft surgery to treat her critical coronary artery disease.

**Outcomes:**

Intra-operatively, internal mammary arteries were not amenable to harvesting due to very dense mediastinal adhesions. Therefore, saphenous vein grafts were performed to the left anterior descending, distal left circumflex, obtuse marginal and distal right coronary arteries. The patient made a satisfactory in-hospital recovery, and was subsequently discharged back to her local hospital for rehabilitation.

**Lessons:**

Patients successfully treated with mediastinal radiotherapy require careful long-term follow-up for the assessment of radiation-induced coronary artery disease. Importantly, mediastinal irradiation may preclude internal mammary artery utilization, and thus alter the strategy for surgical myocardial revascularization.

## 1 Introduction

Coronary artery disease exclusively, affecting the coronary ostia is a rare phenomenon, with an angiographic incidence of 0.07–0.09 %.^[1]^ It is strongly associated with previous mediastinal irradiation, which nowadays is a common treatment modality for specific cancers. We here report the case of a 73-year-old woman who presented with isolated critical stenoses of the coronary ostia, and acute myocardial infarction, without any pre-existing cardiovascular comorbidities.

## 2 Case presentation

**Figure 1:**
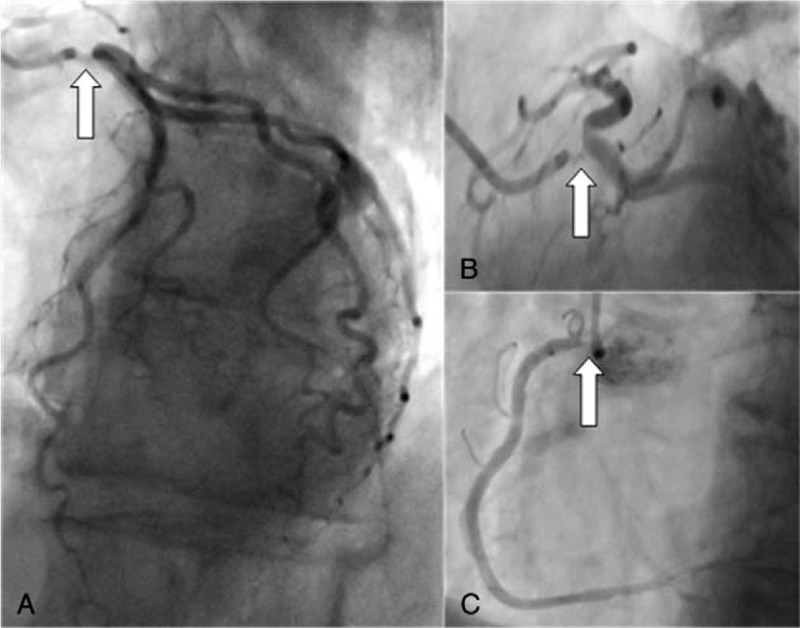
Pre-operative coronary angiogram demonstrating severe ostial lesions (arrows) of the left mainstem (A and B), and right (C) coronary arteries with sparing of the arteries distally.

This study was approved by our hospital ethics committee. A 73-year-old Caucasian woman without other cardiovascular risk factors was admitted to our institution with acute coronary syndrome. Her past medical history was significant for left breast carcinoma treated by mastectomy with lymph node dissection followed by mediastinal radiotherapy 30 years ago. Clinical examination was unremarkable except for a well-healed left mastectomy scar. Admission electrocardiogram demonstrated inferior ischaemic changes. Coronary angiography revealed 99 % –75 % stenoses of the left main stem (Fig. 1A and B, arrows), and right coronary ostia (Fig. 1C, arrow), respectively, with no additional downstream coronary lesions. Trans-thoracic echocardiography demonstrated preserved left ventricular systolic function with ejection fraction 62 %, and no additional valvular abnormalities were detected. An intra-aortic balloon pump was inserted in the context of ongoing chest pain, and critical coronary anatomy. Surgical myocardial revascularization was urgently planned.

Intra-operatively, both internal mammary arteries were very densely adherent to the chest wall, precluding their utilization as conduits. On-pump coronary artery bypass surgery was therefore performed with reversed long saphenous vein bypass grafts to the left anterior descending, obtuse marginal, distal left circumflex, and distal right coronary arteries. Intra-operative Doppler probe scanning confirmed excellent graft flow. Cardiopulmonary bypass was weaned with minimal inotropic support, and intra-aortic balloon counter-pulsation was discontinued 24 hours later. Post-operatively, the patient required a tracheostomy to facilitate weaning from the mechanical ventilator. She was eventually discharged back to her local hospital for physical rehabilitation.

## 3 Discussion

Involvement of the coronary ostia in coronary artery disease is an uncommon pathology occurring in 0.13–0.8 % of angiographic studies, whilst isolated coronary artery stenosis is rarer still at 0.07–0.09 %.^[1]^ Besides atherosclerosis, the aetiology of isolated ostial coronary artery stenosis includes syphilis, Takayasu's arteritis (TA), aortic valve disease, familial hypercholesterolaemia, fibro-muscular dysplasia, iatrogenic trauma from catheter insertion, and mediastinal irradiation.^[2]^

Whilst mediastinal irradiation is a well-established treatment modality for many malignant tumors, it may be complicated by cardiac disease manifesting as pericarditis, valvular dysfunction, conduction abnormalities, myocardial fibrosis, and coronary artery disease, the latter being diagnosed at a mean of 16 years following exposure.^[3–5]^ With recent advances in radiotherapy enhancing survival in certain malignancies, a specific sub-group of patients is now emerging with a delayed onset of coronary artery disease, which may portend an equally serious prognosis.

Radiation-induced coronary lesions commonly involve the ostia, with the right being predominantly affected, and proximal segments with relative sparing of downstream vessels, which usually lie external to the radiation field.^[4]^ The incidence is greater in females.^[4]^ The patho-physiological processes underlying radiation-induced coronary artery disease appear distinct from those of atherosclerotic plaque formation, with evidence of diffuse trans-mural fibrosis, loss of medial smooth muscle cells, and minimal extra-cellular lipid deposition.^[6]^ However, whilst patients with radiation-induced coronary artery disease have few, if any, traditional cardiovascular risk factors, accelerated atherosclerosis may occur if risk factors such as hypercholesterolaemia are present.^[4]^

In the present case, we attributed our patient's coronary ostial lesions to her previous mediastinal irradiation. This is supported by her lack of conventional acquired cardiovascular risk factors such as hypertension, hypercholesterolaemia, diabetes mellitus, or tobacco consumption. Her gender, appropriate history of mediastinal radiotherapy 30 years ago, and the pattern of her coronary anatomy with exclusive ostial involvement further support our hypothesis.

Coronary artery bypass surgery represents the gold standard treatment for ostial coronary artery disease, ideally utilizing bilateral internal mammary arteries to afford these generally, younger patients the prognostic benefit of complete myocardial revascularization in combination with superior graft patency outcomes. Furthermore, a skeletonized harvesting technique may be employed to maximize available conduit lengths. However, the internal mammary arteries may be damaged by their inclusion within the radiation field as part of historic radiotherapy regimes. Extensive chest wall adhesions prohibited safe harvesting of the internal mammary arteries in our patient. Due to her urgent presentation, computed tomography scanning could not be performed pre-operatively, although this might have provided useful insight into the patency, and caliber of the internal mammary arteries, as well as revealing additional radiation-associated sequelae, such as pulmonary fibrosis, and retro-sternal adhesions. Pre-operative angiography can also determine internal mammary artery patency, and comprises an important aspect of surgical strategy planning. Owing to concerns regarding peri-operative internal mammary artery graft flows, we opted to perform saphenous vein grafts for coronary artery bypass.

## 4 Conclusions

Patients who have received mediastinal irradiation at therapeutic doses are at risk of delayed-onset radiation-induced coronary artery disease, which classically manifests with severe ostial lesions. This important sub-group of patients may not present with traditional cardiovascular risk factors, and thus may be overlooked in routine screening pathways. We recommend careful long-term follow-up of post-radiotherapy patients with annual echocardiographic assessment in conjunction with functional evaluation of myocardial ischaemia.

## Author contributions

**Conceptualization:** M.N. Acharya.

**Supervision:** A.-F. Popov.

**Writing – original draft:** M.N. Acharya.

**Writing – review & editing:** M.N. Acharya, M. El-Diasty, B. Schmack, A. Weymann, A. Mansur, A.-F. Popov.
